# Evaluation of medication adherence in hypertensive patients and influential factors

**DOI:** 10.12669/pjms.344.14994

**Published:** 2018

**Authors:** Selma Boratas, Hulya Firat Kilic

**Affiliations:** 1 Selma Boratas, M.Sc. Staff Nurse, Akdogan Health Center, Famagusta, North Cyprus, via Mersin 10 Turkey; 2 Hulya Firat Kilic, PhD, RN. Assistant Professor, Department of Nursing, Faculty of Health Sciences, Eastern Mediterranean University, Famagusta, North Cyprus, via Mersin 10 Turkey

**Keywords:** Adherence, Hypertension, Medication, Patients

## Abstract

**Objective:**

This study aims to evaluate medication adherence in hypertensive patients and to identify the influential factors.

**Methods:**

This descriptive, cross-sectional study included a total of 147 hypertensive patients who were admitted to Akdogan Health Center between December 2016 and February 2017. The Descriptive Data Form and Medication Adherence Self-Efficacy Scale were used as the data collection tools.

**Results:**

The mean Medication Adherence Self-Efficacy Scale score was found to be 70.29 ± 8.52. Age, duration of HT, the frequency of follow-up visit for HT, the status of taking medication regularly, the frequency of blood pressure measurement and the status of alternative method use were found to be effective in medication adherence.

**Conclusions:**

Our study results suggest that medication adherence is good in patients with hypertension (HT). Factors which are effective in medication adherence should be taken into consideration, when evaluating the medication adherence in patients with HT.

## INTRODUCTION

Hypertension (HT) is a major public health problem worldwide, and although it is preventable and controllable, its prevalence has been increasingly rising.^1^,^2^ Hypertension ranks the first among the preventable causes of death worldwide.^3^ The number of patients with HT continues to increase in less developed and developing countries.^4^ While awareness about HT and treatment and control are relatively low worldwide, there are significant differences among countries.^5^

Hypertension may not cause symptoms for a long time and its significant side effects may occur after years. In the absence of symptoms, accepting the treatment by the patient is difficult. Antihypertensive drug therapy is the key method for long-term control of blood pressure.^6^ Therefore, the adherence of the patient is of utmost importance in the treatment of HT.^7^ In the fight against HT, and the low adherence with the prescribed treatment is a major obstacle for achieving the targeted blood pressure control. Individuals with low medication adherence have a high risk in terms of uncontrolled blood pressure and adverse outcomes that may arise.^2^ It has been proven that involvement of patients in decision making, and taking disease and treatment seriously by the patients affects the medication adherence positively.^7^

Adherence is a dynamic process involving the use of the medication at the prescribed frequency and dose.^8^ Adherence to disease in hypertensive patients involves patient’s regular use of medications, adherence to their diet and executing other lifestyle changes. Positive lifestyle changes and medication adherence significantly prevent complications in hypertensive patients. With the knowledge, support and supervision of nurses, the non-adherence of patients to disease and medication decreases significantly, and the level of public health increases.^9^

Nurses should be informed about the benefits of simplifying the medication regimen, performing arrangements of treatment regimen with patients supplying medicines before ended and adherence to medication regimen.^7^ Hacihasanoglu and Gozum (2011) reported that the patient adherence is the most important factor affecting effective blood pressure management in Turkey and in the world, and they provided significant and positive developments in medication adherence, HT management, healthy lifestyle behaviors and body mass index in an interventional study in which they performed patient education and home observation.^10^

This study aims to evaluate medication adherence in hypertensive patients and to identify the influential factors. It is considered that the results obtained about the medication adherence and the affecting factors in patients with HT would guide in the nursing profession which play an effective role in patient care and treatment, in nursing education practices and in clinical studies. It has been suggested that the evaluation of the adherence to medication and the influential factors in patients with HT would present data for the solution of the problem to the individuals who would conduct further studies about this issue. Therefore, the results would directly increase the quality of the patient care and would contribute to public health.

## METHODS

A total of 147 hypertensive patients who were admitted to the Akdogan Health Center between December 2016 and February 2017 were included in this descriptive, cross-sectional study. Voluntary patients who had at least six months of HT diagnosis and were 18 years of age or older were included in the study. The data of the study was collected by the Descriptive Data Form (age, sex, duration of disease, frequency of control etc.) and Medication Adherence Self-Efficacy Scale.

### Medication Adherence Self-Efficacy Scale (MASES)

MASES was developed by Ogedegbe G et al. (2003), and the validity and reliability study for the Turkish population was conducted by Hacıhasanoglu and Gozum in 2005.^10^,^11^ MASES is used to determine the level of Medication Adherence Self-Efficacy in hypertensive patients. MASES, which questions the factors affecting the regular use of antihypertensive drugs of the patients, consists of 26 questions and evaluates the self-efficacy level of the individual in the participation to this statement. The scores between 26 and 78 are obtained from the scale. The increase in the scale score indicates that the adherence of individual to antihypertensive drug treatment is good. The Cronbach alpha value of the scale was found to be 0.92 in the reliability study for the Turkish population by Hacıhasanoglu and Gozum.^10^ In our study, the Cronbach alpha value of the scale was found to be 0.94.

Before starting to receive information from patients with HT, the purpose of study and the duration of questionnaire were explained to the patients by the researcher and a written consent was obtained from the patients. The patients who agreed to participate in the survey were asked to fill out the data collection forms.

Statistical analysis was performed using the SPSS version 20.0 software (IBM Corp., Armonk, NY, USA). Descriptive statistics were expressed in frequency and percentage. Variables showed no normal distribution, and non-parametric techniques were used in the analysis of data. The Mann-Whitney U-test was used to compare the two groups, while the Kruskal-Wallis H test was used to compare more than three groups.

Before conducting this study, a written permission was obtained from the Scientific Research and Publication Ethics Board (Decision No: 2016/34-11), Ministry of Health Basic Health Services Directorate and the patients participating in the study. The study was conducted in accordance with the principles of the Declaration of Helsinki.

## RESULTS

Of the patients included in the study, 44.2% were 61 years and older, 66.7% were female, 83.7% were married and 45.6% were primary school graduate.Table-I In 38.1% of patients, HT duration was between 2-6 years and 70.7% of them used to continue follow up visit once a month. Of the participants, 70.1% were familiar with the name of the medication they used, 69.4% took medication once a day, and 91.2% took it regularly. A total of 72.8% of the patients did not use an alternative method, and only 29.3% of them received education on medication use (Table-II). The overall mean score of MASES for patients(n:47) with HT was found to be 70.29 ± 8.52 (min=30, max=78).

**Table-I:** Comparison of the descriptive characteristics of patients with medication adherence (n=147).

	N (%)	Mean	Statistical Analysis
Age			
27-45	27(18.4)	49.56	X^2^:16.68, SD:2, P:0.000
46-60	55(37.4)	69.49	
61 years and older	65(44.2)	87.97	
Sex			
Female	98(66.7)	75.12	U : 2291, p: 0.650
Male	49(33.3)	71.16	
Marital Status			
Married	123(83.7)	75.20	U : 1329, p: 0.439
Single	24(16.3)	67.88	
Educational Status			
Literate	22(15.0)	84.05	X^2^:7.24, Sd:4, p: 0.124
Primary School	67(45.6)	79.57	
Secondary School	14(9.5)	71.68	
High School	34(23.1)	59.06	
University	10(6.8)	66.30	

**Table-II:** Comparison of medication adherence with characteristics of disease and medication use (n=147).

	N (%)	Mean	Statistical Analysis
***Duration of HT***			
6-12 months	14(9.5)	47.68	X^2^: 8.30 SD= 3 p: 0.040
2-6 years	56(38.1)	73.63	
7-10 years	30(20.4)	70.97	
11 years and over	47(32)	84.21	
***Frequency of follow-up visit for HT***			
Once a month	104(70.7)	79.16	U: 1699 p: 0.022
Once every two months and over	43(29.3)	61.51	
***Knowing the name of the medication used***			
Knowing	103(70.1)	74.57	U: 2207 p: 0.802
Not knowing	44(29.9)	72.66	
***Frequency of medication intake***			
Once a day	102(69.4)	71.30	U: 2027,5 p: 0.258
Twice a day and over	45(30.6)	79.94	
***Status of taking medication regularly***			
Yes	134(91.2)	77.88	U: 350.5 p: 0.000
No	13(8.8)	33.96	
***Status of alternative method use***			
Yes	40(27.2)	63.49	U:1719.0 p: 0.050
No	107(72.8)	77.93	

When the mean scores of MASES were examined according to the age of patients participating in the study, MASES scores of hypertensive patients aged 61 years and older were found to be higher than those of the other age groups (p: 000). The difference between the mean scores of MASES of patients and sex (p: 0.650), marital status (p: 0.439), and educational status (p: 0.124) was not statistically significant (p>0.05).

The difference between the mean “duration of disease” and the mean scores of MASES in hypertensive patients was statistically significant (p<0.05), and the mean scores of MASES of patients with the disease duration of 11 years and over were found to be higher than the other groups (p:0.004). When the mean scores of MASES were analyzed according to “Status of Continuing Follow-up Visit”, the scores of MASES of patients who continue follow-up once a month were found to be higher than the patients who continue follow-up Every Two Months And Over (p<0.05).

While there was no significant difference between the mean scores of MASES and the status of knowing the medication used by hypertensive patients (p: 0.802), and “the status of receiving education on medication use” (p: 0.337), the mean scores of MASES of patients who took medication regularly were higher than the scores of those who did not take regularly (p: 0.000).

The mean scores of MASES of hypertensive patients according to the “Status of Alternative Method Use” showed statistically significant difference, and the MASES scores of the patients who did not use alternative method were higher (p: 0.050) (Table-II).

## DISCUSSION

The level of mean MASES score of hypertensive patients was found to be good in this study (70.34 ± 8.60) (range: 30 to 86). While there are study results reporting good levels of medication adherence in patients with HT similar to our results^7^,^12^-^14^, the study results showing moderate and low levels of medication adherence in hypertensive patients are also present.^6^,^15^ It may be considered that regular admission of patients to the primary health care in our study group, not using alternative treatment in the majority of them and taking medications regularly positively affected medication adherence.

There are many factors that affect medication adherence of hypertensive patients. In the literature, there are different results regarding the relationship between medication adherence and age.^7^,^12^ It can be considered that the difference in the results of studies may arise from the differences of problems caused by the disease according to age. In addition, the complications of disease increase with an increase in age, or the effect of a condition that disrupts body image is more prominent in youth could be the main causes of the differentiation of study results. While there was no difference between age and medication adherence in the study performed by Hacıhasanoglu^7^, Cingil^16^ showed that younger age affected medication adherence positively. In our study, we found that medication adherence of hypertensive patients aged 61 years and older was better than the other age groups (p<0.05, Table-I). The increase in adherence with age is an expected outcome for our study. Since the patients adopt diagnosis by age, the adherence to regular use of medications improves. It is thought that the difficulty in adopting the diagnosis of disease in younger people and the fact that they are not aware of the importance of regular medication use may have influenced the result of the study.

Newly diagnosed hypertensive patients often show less stability in taking medications.^13^ According to our study, the medication adherence was found to be higher in patients with ’HT duration’ of 11 years and over (p<0.05, Table-II). In some studies with hypertensive patients, although there was no relation between duration of HT and medication adherence^9^,^13^,^17^, Tumer A et al. (2016) observed that medication adherence increased, as the duration of HT increased, similar to our study.^17^ It is an expected outcome for our study that increase in medication adherence as the duration of HT increases, since studies are in the majority that supporting the adherence is low in the beginning of diagnosis, and the behaviors of adopting and adherence are observed as the duration of diagnosis increases.

HT complications are at the top causes of death in adults. Complications of HT such as heart failure, coronary heart disease, peripheral vascular disease, renal failure, brain, eye and hypertensive crisis make life considerably difficult in the adult age group.^18^ For diagnosis and treatment of HT, blood pressure measurement and follow-up should be done correctly. In the study on diabetes patients by Kaymaz and Akdemir (2016), the adherence of individuals with a follow-up frequency of 4-12 months was found to be better than the individuals with a follow-up frequency of over one year.^19^ Similarly, in our study, the patients with more frequent follow-up visits for HT were found to have a higher adherence (p<0.05, Table-II). In contrast to our study findings, there was no significant difference between follow-up frequency and medication adherence in the study by Gun and Korkmaz.^18^ Regular follow-up of patients is important for controlling Blood Pressure and for preventing complications by providing the disease monitorization. It has been suggested that regular follow-up of patients would allow them to see alterations in blood pressure and, thus, they would cooperate with health professionals for the treatment and this would have an impact on medication adherence.

Taking medications regularly in hypertensive patients affects medication adherence positively. In our study, medication adherence was found to be higher in patients taking HT medications regularly (p<0.05). In the study conducted by Hacihasanoglu (2009), the relationship between the status of taking medication regularly and medication adherence was found to be statistically significant, as similar to our study findings. Patients should be informed about not to give up medication and not to skip a dose if the side effects of medication develops in the education of medication adherence of patients about taking medications regularly.

It is known that hypertensive patients use alternative treatment methods to control blood pressure.^20^ In a study conducted by Guven SD et al. (2013), to investigate the use of complementary treatment by patients with HT in Turkey, they found that 52.7% of patients were using alternative methods.^21^ Cevheroglu and Cagliyan (2016) found a significant negative correlation between medication adherence and the use of alternative methods in hypertensive patients in northern Cyprus.^14^ In the study conducted by Anadol and Discigil (2009), it was observed that the patients who failed in adherence to medication tended to use alternative methods more frequently.^22^ In our study, medication adherence was found to be higher in patients who did not use Alternative Method to Control Hypertension (p≤0.05,). In the study by Boima V et al. (2015), which is similar to our study findings, the adherence of patients who did not use alternative method was found to be better.^23^ Considering the literature studies, the higher adherence of patients who do not use alternative therapy is an expected result for our study.

### Limitations

This study is limited with hypertensive patients who admitted to one health center in Northern Cyprus only. Therefore, the results can be generalized only to this population.

## CONCLUSIONS

Our study results showed that the medication adherence of hypertensive patients included in the study was found to be satisfactory. It was also found that age, duration of HT, taking medication regularly and use of alternative methods were the factors leading to differences in the adherence levels of the patients to the medication. To achieve success and improve adherence to medication in hypertensive patients, further informative educations should be given about the disease of HT and its complications, particularly to younger patients. Based on these results, we suggest that hypertensive patients should be informed about the careful use of alternative methods, considering the fact that patients who do not use alternative methods for HT control have a higher medication adherence.
